# Ion energy spectra directly measured in the interaction volume of intense laser pulses with clustered plasma

**DOI:** 10.1038/s41598-018-27665-x

**Published:** 2018-06-20

**Authors:** S. G. Bochkarev, A. Faenov, T. Pikuz, A. V. Brantov, V. F. Kovalev, I. Skobelev, S. Pikuz, R. Kodama, K. I. Popov, V. Yu. Bychenkov

**Affiliations:** 1 P.N. Lebedev Physical Institute, RAS, Moscow, 119333 Russia; 2 Open and Transdisciplinary Research Initiatives, Osaka University, Suita, Osaka 565-0871 Japan; 3 Joint Institute for High Temperatures, RAS, Moscow, 125412 Russia; 4 Graduate School of Engineering, Osaka University, Suita, Osaka 565-0871 Japan; 5 Center for Fundamental and Applied Research, VNIIA, ROSATOM, Moscow, 127055 Russia; 6 Keldysh Institute of Applied Mathematics, RAS, Moscow, 125047 Russia; 7 National Research Nuclear University MEPhI, Moscow, 115409 Russia; 8 Institute of Laser Engineering, Osaka University, Suita, Osaka 565-0871 Japan; 9 University of Ottawa, Ottawa, ON K1N 6N5 Canada

## Abstract

The use of gas cluster media as a target for an intense femtosecond laser pulses is considered to be uniquely convenient approach for the development of a compact versatile pulsed source of ionizing radiation. Also, one may consider cluster media as a nanolab to investigate fundamental issues of intense optical fields interaction with sub-wavelength scale structures. However, conventional diagnostic methods fail to register highly charged ion states from a cluster plasma because of strong recombination in the ambient gas. In the paper we introduce high-resolution X-ray spectroscopy method allowing to study energy spectra of highly charged ions created in the area of most intense laser radiation. The emission of CO_2_ clusters were analyzed in experiments with 60 fs 780 nm laser pulses of 10^18^ W/cm^2^ intensity. Theory and according X-ray spectra modeling allows to reveal the energy spectra and yield of highly charged oxygen ions. It was found that while the laser of fundamental frequency creates commonly expected monotonic ion energy spectrum, frequency doubled laser radiation initiates energy spectra featuring of distinctive quasi-monoenergetic peaks. The later would provide definite advantage in further development of laser-plasma based compact ion accelerators.

## Introduction

During the past decades, the interaction of laser radiation with nanostructured media (clusters, nano-wires, nano-spheres, carbon nano-tubes, snow targets and etc.) has attracted much attention in the context of studying fundamental properties of matter under extreme conditions^1–26^. Cluster plasma offers a potential for various applications, including nuclear fusion reactions^2,27–30^, laser-driven high-energy ion^1,31–38^ and electron sources^39–41^, and X-ray and betatron emission^42–50^. The benefits of cluster targets include not only the possibility of efficiently generating various particle and radiation beams, i.e., high-energy electron and ion beams, bright flashes of X-rays and radioactivity, but also the absence of debris that can damage optics and a quick renewal of the initial target parameters after each laser action. Cluster targets are also unique because of the possibility of attaining a high rate of absorbing laser radiation up to the total, which can boost further potential applications.

We note that despite about 20 years of intensive investigations, a complete understanding of the interaction of short laser pulses with clusters is still far from clear. For example, the formation of very pronounced peaks in the contour of the OVIII Ly_*α*_ line emitted by a CO_2_ cluster plasma created by the second harmonic of Ti:Sa laser light with an intensity of the order of 10^18^ W/cm^2^ was observed^32^. These X-ray spectra features have not yet been explained during practically 20 years. This paper is intended to contribute to understanding the observed spectroscopic features. The key supposition that could explain them is the generation of quasimonoenergetic high-*Z* oxygen ions of different energies, and we follow this direction below. We note that quasimonoenergetic protons were recently observed when an intense laser pulse irradiated a nanosized water spray. This was interpreted in terms of the contribution of many Coulomb exploding clusters in a focal volume to produce a peaked spectrum of accelerated protons^51^. Nevertheless, highly charged oxygen ions were not detected in this experiment in measurements with a Thomson parabola because of fast oxygen recombination as a result of propagation inside the spray. Similar was the case in the experiment by F. Abicht *et al*.^52^, where water droplets were irradiated by powerful laser radiation at intensities up to 5 · 10^19^ W/cm^2^ and no highly charged carbon and oxygen ions were observed with the Thomson parabola technique. This situation is typical for cluster targets^51–55^. Recombination in the surrounding gas leads to a production of neutrals and negative ions, which is well detected in laser–cluster experiments^54,55^.

There are many direct methods for registering ions, such as time-of-flight measurements with a microchannel plate detector (MPD), radiochromic film (RCF)^56^, CR-39 track detector^57^, and Thomson parabola^58^. However, those conventional methods fail for characterization of ion enery spectrum in cluster plasma, as they utilize measurements outsize of the interaction volume. Although the X-ray spectral method has already been used to detect multicharged ions some time ago^10,32^, it is still not widely used. We believe that this method can be applied for registration of high-charge-state ions in the future for relevant experiments similar to such as in refs^51–55,59,60^. The standard methods mentioned above require measurements outside of the interaction region (e.g., with Thomson parabola) and are not able to diagnose the presence of highly charged ions due to electron capture in an ambient medium. Ultimately, even while the developed method utilizes quantitative approach (see Sec. Methods for details), it is the only one available till now to measure high-charge-state ions inside the interaction volume. In our experiments, we used a spectroscopic measurement that allowed registering highly charged oxygen states and reconstructing high-*Z* ion spectra. Our aim is to use numerical simulations and analytic theory to interpret these experiments and prove that the X-ray spectroscopic method for measuring the ion spectrum is well complementary to the traditional methods and has an advantage for a large-volume gas target.

It is well known that there are two basic regimes of plasma expansion depending on the ratio between the electron Debye radius *λ*_D_ and the cluster diameter *D*: quasineutral expansion (or close to it), where ${\lambda }_{{\rm{D}}}/D\ll 1$ ^61–64^, and cluster explosion with strong charge separation (Coulomb explosion in the limit case *λ*_D_ → ∞), where ${\lambda }_{{\rm{D}}}/D\gg 1$ ^21,51,65^. These regimes are very well studied for short (femtosecond) energy input because simplified theories are now well developed for small and large ratios *λ*_D_/*D*. They are relevant to either low (typically, subrelativistic) or ultra-high laser intensities. But the intermediate regime with $D\gtrsim {\lambda }_{{\rm{D}}}$ is theoretically more complicated and requires numerical modeling. Our experimental results for moderate laser intensities ${I}_{{\rm{L}}}\simeq {10}^{18}$ W/cm^2^ satisfy this condition. The interaction of laser pulses with cluster targets in this regime is also relevant for laser-triggered thermonuclear reactions as possible neutron sources.

To interpret the experimental data, we here adapt a numerical approach for studying a kinetic collisionless expansion of multispecies plasma clusters in a self-consistent electric field driven by laser-heated electrons^66^ and the theory of the quasineutral adiabatic expansion of a hot macroscopic gas plasma^61,63,64^ of the focal volume. We use both a simplified electrostatic kinetic numerical model and PIC (particle-in-cell) simulations below to model cluster expansion in the case where an ultrashort second-harmonic laser pulse interacts with CO_2_ clusters. Namely, in the case of 2*ω* interaction, the laser contrast suffices for clusters to intact, while in the 1*ω* case, the clusters are expected to be significantly destroyed. Correspondingly, ion acceleration results from the focal hot plasma expanding as a whole in the 1*ω* case. Because the laser hot spot has a cylinder-like shape in our experiment, this is predominantly radial expansion. Given the general impossibility of directly measuring highly charged state ions from cluster targets using conventional methods, we here present a theoretical background for measuring highly charged ions indirectly with high-resolution X-ray spectroscopy. Interpreting spectra using the developed theory and numerical simulations clearly show the prospects for such a diagnostic tool.

## Experimental Setup and Diagnostics

**Figure 1:**
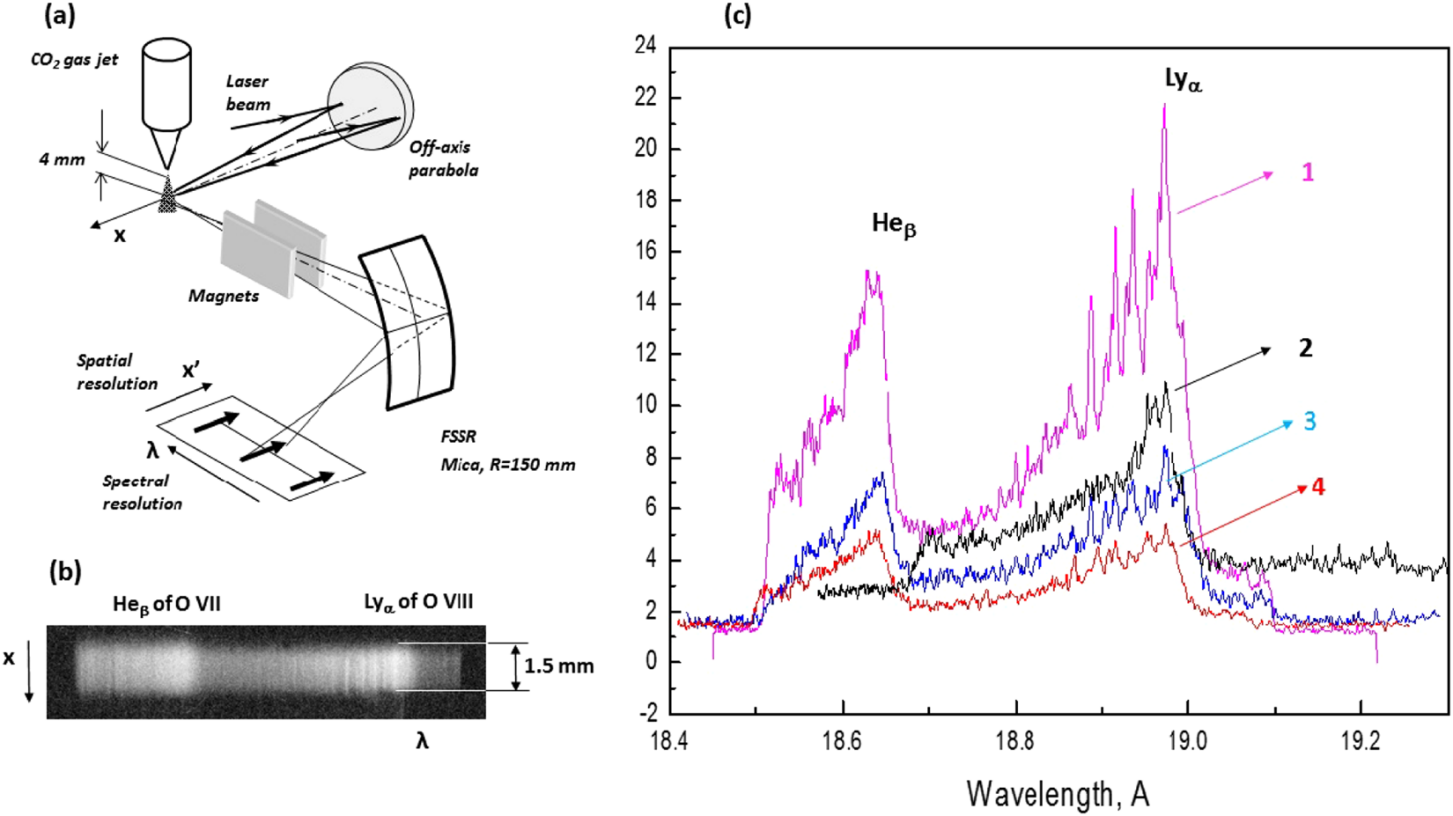
(**a**) Scheme of experiment. (**b**) Image of typical spectrogram. (**c**) Traces of X-ray spectra of Ly_*α*_ of OVIII obtained under different experimental conditions (also see Table 1). Curves 1–4 correspond to the parameters in Table 1.

The experiments^32^ were performed on the laser facility at the Center d’Etudes de Saclay (France). The experimental scheme is shown in Fig. 1a. A titanium–sapphire laser with a 60 fs pulse duration and energy up to 70 mJ at the fundamental frequency (*λ*_L_ ≈ 0.8 *μ*m) or up to 20 mJ at the second harmonic (*λ*_L_/2 ≈ 0.4 *μ*m) was used to heat the cluster target. Focusing the radiation with a parabolic mirror gave a radiation flux density up to 10^18^ W/cm^2^ in a focal spot of $\simeq 5\,\mu {\rm{m}}$ diameter. The cluster target was produced by adiabatic expansion into vacuum of a small amount of CO_2_ gas emerging from a gas valve through a pulsed nozzle 0.3 mm in diameter. The gas pressure in the valve was varied from 10 to 40 bar. The X-ray radiation of the plasma was detected by a focusing spectrometer with spatial resolution (FSSR)^67,68^ with a spherically bent mica crystal with a curvature radius *R* = 150 mm. The spectral resolution of the spectrometer was *λ*/*δλ* ~ 3000 with a spatial resolution ~20 *μ*m. The spectral range covered by the spectrometer was 18.5 to 19.2 Å, which allowed observing the 1*s*3*p*^1^*P*_1_ − 1*s*^2^ (*n* > 3) line of He-like OVII and the 2*p*^2^*P* − 1*s*^2^*S* Ly_*α*_ line of H-like OVIII. An example of the spectrograms and densitometer traces obtained by heating clusters with laser pulses of the first and second harmonic of the Ti:Sa laser are shown in Fig. 1b. The laser and CO_2_ gas parameters used in the experiments are presented in Table 1. It was previously found^32^ that the X-ray spectra of H-like oxygen have very unique structures (see Fig. 1b and c) in the shapes of the emission lines when CO_2_ clusters are heated by 2*ω* radiation of a Ti:Sa laser with intensity of about 10^18^ W/cm^2^.

**Table 1:** Laser and cluster target parameters used in the experiment.

N	Laser wavelength, nm	Laser energy, mJ	Gas pressure, Bar	Comments
1	400	16	45	Strong features in the spectrum
2	800	70	15	Practically no features in the spectrum
3	400	18	40	Some features in the spectrum
4	400	18	30	Very weak features in the spectrum

Here, N corresponds to the numbered curves in Fig. 1c.

## Results

Two main characteristic features of the emission spectra of a CO_2_ plasma can be seen in Fig. 1b and c. The emission spectra of a plasma produced by the interaction of a femtosecond laser pulse at the fundamental frequency (*λ*_L_ ≈ 0.8 *μ*m) with CO_2_ clusters clearly show the presence of a strong asymmetry of the profile of the spectral lines of H- and He-like oxygen ions. The same asymmetry is also seen in the case where the second harmonic of Ti:Sa radiation with *λ*_L_/2 ≈ 0.4 *μ*m is used. This feature is due to the presence of a strong asymmetry of the profiles of the spectral lines of H- and He-like oxygen ions. Such an asymmetric line shape cannot be due to thermal Doppler or Stark broadening of the spectral lines in plasma. A simple model was proposed^32^ that explains the observed shape of the spectral lines. The main fundamentals of such model are (i) line broadening due to macroscopic motion (expansion) of the cluster plasma and (ii) appearance of asymmetry of the shapes of the lines of oxygen ions as a result of the photoionization absorption on H- and He-like carbon ions present in the CO_2_ plasma. Nevertheless, the second feature of the emission spectra the appearance of multiple clearly resolved peaks at the observed spectral lines and, in particular, at the shape of the Ly_*α*_ of H-like oxygen emitted by CO_2_ cluster plasma created by a pulse at the second harmonic of the Ti:Sa laser has not yet been explained. The experiments do not show such clearly pronounced peaks for the laser pulse at the fundamental frequency. Below, we explain the different spectral features in the cases of 1*ω* and 2*ω* radiation and the nature of the deep modulation of the *O*^+8^ ion spectrum from a cluster plasma heated by the second harmonic.

**Figure 2:**
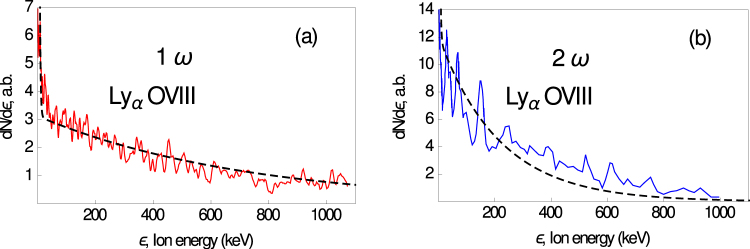
Reconstructions of Ly_*α*_ of H-like oxygen energy spectra for (**a**) 1*ω* and (**b**) 2*ω* radiation of Ti:Sa shown in red and blue. The dashed black curves are the theoretical modeling.

First, we note that because the spectral line broadening in our experiments was due to the directed motion of plasma particles, the analysis of the line profiles can be used to measure the distribution of ions with respect to the expansion velocity, i.e., distribution of the number *N*(*ε*) of ions with respect to their energy *ε*. It can be seen that the experimental spectra clearly show two slopes corresponding to two different ion temperatures for low and high ion energy ranges. In Fig. 2, we show the reconstruction of the shapes of the Ly_*α*_ of H-like oxygen spectra (see Methods for details) emitted by CO_2_ clusters irradiated by 1*ω* and 2*ω* Ti:Sa laser pulses. It is important that our spectroscopy method allows reproducing ion spectra directly in the laser–plasma interaction region, which is not often possible with other methods.

### Theoretical description and comparison with experiment

To treat the experimental results theoretically, we use models based on the analytic 1D solution of the kinetic equations for electrons and ions of a hot plasma column radially expanding in a quasineutral manner, a simplified electrostatic PIC method for a spherically symmetric cluster with a given initial electron distribution, which models a short laser pulse, and full 3D PIC simulations of the laser–cluster interaction. These approaches are not intended to be all-inclusive, i.e., to include the effect of a finite laser contrast ratio (prepulse). They model only the main pulse interaction with a plasma target, and the prepulse is taken into account by modifying the initial target density profile. Note, that it is hardly possible to obtain quantitative agreement from comparison of theoretical model with experimental results, because the number of clusters in the focal volume, distances between them, their maximum density and density profiles are not well predictable. However, that is in fact the first explanation of high-charge-state ion energy distribution for cluster plasma.

As a starting point, we assume that clusters in a gas are not fully destroyed as a result of the laser prepulse in the case of 2*ω* laser pulse radiation, because the 2*ω* radiation has much better contrast than the 1*ω* radiation. As a result, the laser pulse interaction with clusters produces a spectrum of highly charged state ions with deep modulations due to the contribution of some number of clusters from a focal volume (see Fig. 2b). On the other hand, it is unlikely that clusters survived before main pulse arrival in the 1*ω* case because of the low laser contrast (due to the prepulses and pedestal). We believe that the clusters in this case are significantly or fully destroyed and the propagating laser pulse in fact interacts with gas and strongly heats the plasma in the caustic domain, which evolves in the form of a radially expanding cylindrical multispecies plasma (laser–plasma channel). Obviously, such an expanding macroplasma (unlike the cluster microplasmas) should lead to the formation of a quasimonotonic ion energy spectrum with no deep modulations^63,64^. The corresponding energy spectrum from observation is shown in Fig. 2a.

Therefore, we proceed from the fact that ions are accelerated from a radially expanding plasma column in the 1*ω* case and from both a quasi-homogeneous gas macroplasma of laser caustics and single clusters within it in the 2*ω* case. To describe ion acceleration from the heated gas laser–plasma channel, we use the plasma expansion model^63,64^ in a symmetric cylindrical geometry with adiabatic ion acceleration in the radial direction immediately after the 1*ω* ultra-short (60 fs) laser pulse terminates. In our theoretical model, we assume a two-temperature electron distribution with hot and cold electrons typically observed in laser–plasma interactions. The hot-electron density is typically much smaller than the cold-electron density, but the temperature and pressure of hot electrons are larger than the temperature and pressure of the cold electrons. The plasma expansion is hence mostly determined by the hot electrons. The temperature of laser-heated hot electrons is a key parameter in our model. We estimate it from the standard ponderomotive scaling^69^ ${T}_{{\rm{h}}}=\mathrm{((1}+{a}_{0}^{2}{)}^{\mathrm{1/2}}-\mathrm{1)}{m}_{e}{c}^{2}$, where ${a}_{0}=0.85{\lambda }_{{\rm{L}}}[\mu m]\sqrt{{I}_{{\rm{L}}}{\mathrm{[10}}^{18}\,{\rm{W}}/{{\rm{cm}}}^{2}]}$ is the dimensionless laser field amplitude. For the pulse with the maximum laser intensity of 10^18^ W/cm^2^, this gives *T*_h_ ≈ 100 keV and *T*_h_ ≈ 30 keV respectively for 1*ω* and 2*ω* radiation. Because the laser pulse duration is much shorter than the ion acceleration time, we neglect the direct ponderomotive ion acceleration and regard the ion acceleration as triggered by the temperature effect in the adiabatic plasma expansion regime. For simplicity in our analytic model, we assume that the plasma column contains two ion species (denoted by the subscripts 1 and 2): the ion bulk with the average charge 〈*Z*〉_1_ = 3 and atomic number 〈*A*〉_1_ = 15 and the high-*Z* impurity with 〈*Z*〉_2_ = 8 and 〈*A*〉_2_ = 16. Because the ionization potential of the two inner levels of oxygen exceeds the laser ponderomotive potential, the ions *O*^+7^ and *O*^+8^ should indeed be treated as impurities. Making an insignificant simplification, we neglect the difference between *O*^+7^ and *O*^+8^ and consider a single impurity *O*^+8^. Similarly, we also treat the ions *C*^+5^ and *C*^+6^ as a single impurity. Their charge-to-mass ratio is nearly the same as for *O*^+8^, and we therefore replaced all impurities *O*^+7^, *O*^+8^, *C*^+5^, and *C*^+6^ with the approximate effective impurity with 〈*Z*〉_2_ = 8 and 〈*A*〉_2_ = 16 because only the charge-to-mass ratio is important. We used the approximation of an average ion with 〈*Z*〉_1_ = 3 and 〈*A*〉_1_ = 15 (〈*Z*〉_1_/〈*A*〉_1_ = 1/5) to describe the entire multispecies bulk for oxygen from *O*^+1^ to *O*^+6^ and for carbon from *C*^+1^ to *C*^+4^.

The dynamics of the adiabatic cylindrical plasma expansion is determined by the solution of the kinetic equation system for ions and electrons with initial Maxwellian distributions for all kinds of particles. For a two-ion component plasma with two-temperature electrons (hot and cold components), the spectrum of the light ion impurity can be expressed as^64^

1 $$\frac{dN}{d\varepsilon }\simeq \theta ({\varepsilon }_{{\rm{ch}}}-\varepsilon )C{n}_{{\rm{c}}}\,\exp \,(-\frac{\varepsilon }{{\langle Z\rangle }_{2}{T}_{{\rm{c}}}})+\theta (\varepsilon -{\varepsilon }_{{\rm{ch}}})C{n}_{{\rm{h}}}\,\exp \,(-\frac{\varepsilon }{{\langle Z\rangle }_{2}{T}_{{\rm{h}}}}),$$

where *θ*(*x*) is the unit step function and ${\varepsilon }_{ch}\simeq {\langle Z\rangle }_{2}{T}_{{\rm{c}}}\,\mathrm{ln}\,({n}_{{\rm{c}}}/{n}_{{\rm{h}}})$. We impose the quasineutrality condition 〈*Z*〉_1_*n*_10_ + 〈*Z*〉_2_*n*_20_ = *n*_h_ + *n*_c_, and *C* is a constant that can be found from the normalization condition ${\int }_{0}^{\infty }d\varepsilon \,dN/d\varepsilon ={N}_{20}$, where *N*_20_ is the total number of impurity ions. Equation (1) holds under the natural assumptions ${T}_{{\rm{h}}}\gg {T}_{{\rm{c}}}$, ${n}_{{\rm{h}}}\ll {n}_{{\rm{c}}}$, and ${\langle Z\rangle }_{2}{n}_{20}\ll {\langle Z\rangle }_{1}{n}_{10}$. There is a direct connection between the two-temperature electron distribution and two slopes of the high-*Z* ion energy spectrum. The quasineutrality approximation used is applicable for a hot plasma gas under the experimental conditions because *λ*_*D*_/*L* < 1, where ${\lambda }_{D}=\sqrt{{T}_{{\rm{h}}}\mathrm{/4}\pi {n}_{{\rm{h}}}{e}^{2}}$ and *L* is the characteristic scale of plasma inhomogeneity. The result of theory (1) for the ion impurity energy spectrum is shown by the black dashed curve together with the experimental findings in Fig. 2a. To fit the experimental data for the 1*ω* radiation, we chose the electron parameter relations *T*_h_/*T*_c_ = 300 and *n*_h_ = 0.03*n*_c_, where *T*_h_ ≈ 100 keV in accordance with the ponderomotive estimate given above. Radial expansion of the hot spot gas plasma also well describes the averaged (over spectral oscillations) shape of the *O*^+8^ spectrum in the 2*ω* case, as shown in Fig. 2b (black dashed line). The theoretical curve presented in the figure corresponds to *T*_h_ ≈ 30 keV, *T*_h_/*T*_c_ ≈ 90, and *n*_h_ ≈ 0.1*n*_c_.

The above value of hot electron densities have been taken to achieve best agreement with experimental spectra. In the case of high contrast laser pulse clusters should sustain solid under heating by prepulse, what consistenly results in more effective hot electron generation and 3 times higher electron densities. By modeling for the ratio of OVIII Ly_*α*_ to He_*β*_ line intensities the estimate for bulk cold electron temperature was obtained to be around 140–150 eV. At the same time, the observed spectrum introduces rather weak dependence on hot electron density.

**Figure 3:**
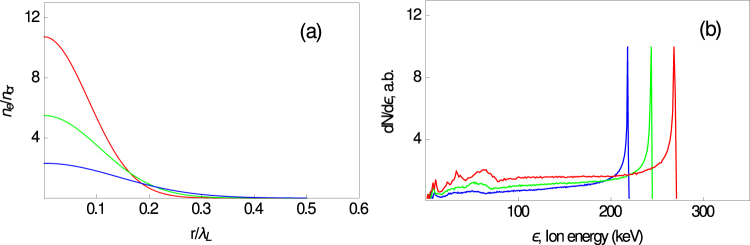
(**a**) The initial electron density distribution (in critical density units ${n}_{cr}={m}_{e}{\omega }_{L}^{2}\mathrm{/4}\pi {e}^{2}$) used in the simulations corresponding to clusters of 160 nm (blue), 120 nm (green), and 100 nm (red) diameter. (**b**) Spectra from EGSPC simulations for impurity ions (for 160, 120 and 100 nm clusters) for the maximum laser intensity *I*_L_ = 10^18^ W/cm^2^.

The nonmonotonic behavior of the *O*^+8^ spectrum in the 2 *ω* case can be explained by ion acceleration from expansions of clusters in the focal volume. We model such cluster expansion by the electrostatic gridless spherical particle code (EGSPC) and by the full 3D relativistic PIC code Mandor. In these simulations, we consider 50 to 160 nm diameter clusters with two ion species, i.e., ions with 〈*Z*〉_1_ ≈ 3 and 〈*A*〉_1_ ≈ 15 as a bulk and small impurity (few percent) of a high 〈*Z*〉_2_/〈*A*〉_2_ ratio, ${\langle Z\rangle }_{2}/{\langle A\rangle }_{2}\simeq \mathrm{1/2}$. We used smoothed density distributions of the clusters in our simulation because we believe that a steplike density profile (ideal clusters) is unlikely to occur even in the 2 *ω* case because of the nonideal contrast ratio at the 1 to 10 ps time scale, similar to the argument in ref.^70^. This is because the ps-prepulse leads to some cluster expansion before interaction with the main pulse. In this context, we assume that the ion cluster density profiles are Gaussian (as shown in Fig. 3a), i.e., $n\sim \exp (\,-\,\mathrm{ln}\,\mathrm{(2)}{r}^{2}/{r}_{0}^{2})$, where 2*r*_0_ is the FWHM cluster size. The energy spectra for the expanding clusters from the EGSPC simulation are shown in Fig. 3b for the 2*ω* pulse with a peak intensity of 10^18^ W/cm^2^. We chose the following electron parameters based on the results of PIC simulations (see below): *T*_h_ ≈ 100, 120, and 140 keV, *n*_*h*_/*n*_*c*_ ≈ 0.05, *T*_c_ ≈ 10 keV for 160, 120, and 100 nm clusters, which contain $\lesssim {10}^{7}$ atoms. We note that the hot electron temperature of clusters could be higher than for a smooth gas macroplasma because of possible resonant heating of clusters. It can be seen that the spectra have a sharp cutoff with a pronounced quasimonoenergetic feature close to the cutoff. These features can be explained as a result of light ions accelerating at the expanding plasma cloud front, similar to what was described in ref.^66^. With this simple electrostatic model, we can take both cluster size dispersion and different initial radial cluster positions in the focal volume into account, reducing electron temperatures that can also be attributed to off-axis positions of clusters with respect to the laser beam axis. This model qualitatively reproduces the characteristic energies of high-*Z* impurity ions accelerated from clusters corresponding to the experimental findings. Thus, in our theoretical modeling we were able to describe spectral peaks that correspond to different clusters (with different size, different densities, and different density profiles smoothing due to the prepulse effect). Because of chaotic appearance of the clusters in a focal volume, exact characterization of these peaks in experiment is impossible and qualitative picture is the only thing that is possible, similar to refs^51,52^ for quasimonoenergetic protons from a water spray. However, as mentioned above, even qualitative explanation of high-Z ion energy distribution allows identify highly charged ions as well as relevant cluster characteristics.

**Figure 4:**
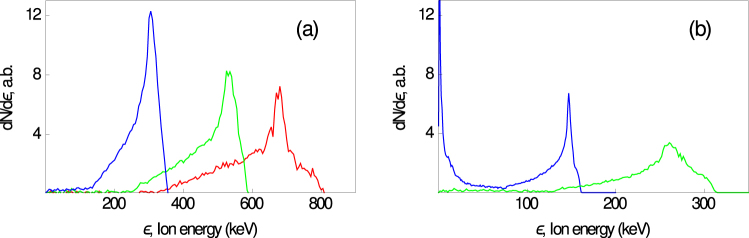
Spectra of impurity cluster ions from the PIC simulations corresponding to density profiles shown in Fig. 3a for (**a**) *I*_L_ = 10^18^ W/cm^2^ and (**b**) *I*_L_ = 3 · 10^17^ W/cm^2^ (**b**).

The results of the 3D PIC simulation are summarized in Fig. 4. Nonmomotonic features can be clearly seen in the spectra: there are quasimonoenergetic distributions with well-pronounced energy peaks (with widths of the order of 100 keV) shifted to the cutoff energies (300 to 800 keV). The characteristic peak energies are proportional to the hot electron temperatures. For cluster diameters of 100, 120, and 160 nm, our PIC simulations give the corresponding hot electron temperatures *T*_h_ ≈ 140, 120, and 80 keV. The electron distributions are two-temperature-like with *T*_*c*_ ≈ 10 to 30 keV. The steeper initial density profile (smaller cluster size), the higher cutoff energy of the spectrum, and the corresponding peak energy agree with the qualitative results of the electrostatic modeling. The reduction of the pulse intensity from 10^18^ W/cm^2^ to 3 · 10^17^ W/cm^2^ results in a decrease of the electron temperature and maximum (cutoff) ion energy by a factor of two. The corresponding energy spectra are shown in Fig. 4 (right panel). The energy range of the oxygen impurity is consistent with the experimental findings including the nonmonotonic features of the ion spectra.

We have thus demonstrated that for the 2*ω* laser pulse interaction with a cluster plasma, the theory reproduces the experimental spectrum of O^8+^ ions in the form of a monotonically decreasing slope (due to gas plasma expansion) of two different steepnesses with peaks (from cluster expansions) on it. Both the electrostatic and the full PIC numerical models show the existence of well-pronounced spectral peaks for moderately smoothed clusters expected in the rather high-contrast 2*ω* experiment. It cannot be excluded that strongly expanded but not entirely destroyed clusters from the prepulse stage could also lead to a weak modulation of the observed ion spectrum in the 1*ω* case (cf. Fig. 2a).

## Discussion

This paper reports theoretical modeling of multicharged ion acceleration from a CO_2_ cluster plasma and justifies the result of an experiment demonstrating unusual features in the X-ray spectra of H-like oxygen observed when a cluster gas is irradiated by the second harmonic of a Ti:Sa laser with intensities about 10^18^ W/cm^2^. The performed theoretical modeling based on a three-dimensional PIC simulation, a simplified electrostatic kinetic model, and an analytic solution of the Vlasov equations allowed proving the nonmonotonic features of the ion spectra, thus providing the first interpretation of the experimental findings since their original publications^32,33^.

We showed that the shape of the spectra of highly charged state ions is defined by particles accelerated from both the gas and clusters. Ions originating from clusters provide pronounced peaks in the spectra for 2*ω* high-contrast radiation. These peaks are very smoothed or even absent for a low-contrast laser pulse, which is the case for laser interaction at the fundamental frequency, where the experiment does not show noticeable spectral modulations. In the cases of both high and low laser contrast (the 2*ω* and 1*ω* cases), the overall integral shape of the spectra for highly charged state ions shows two different steepnesses, identifying two kinds electrons with low and high energies. Finally, we conclude that a theoretical basis for X-ray spectroscopic measurements of ion spectra is now established. We have shown that high-resolution X-ray spectroscopy is an effective tool that allows measuring the spectra of multicharged laser-accelerated ions, even with a very complicated fine structure, with good accuracy.

## Methods

### Spectrum reconstruction

Because the broadening of spectral lines in our experiment is due to plasma motion, the analysis of line profiles can be used to measure the distribution of ions with respect to the expansion energies. Most simply (and precisely), this can be done in the case of an optically thin plasma where photoionization absorption is absent and the profile *F*_*ik*_ (*ω* − *ω*_*ik*_) of a spectral line resulting from the radiative *i* − *k* transition is directly proportional to the distribution of ions with respect to the velocities:

2 $${F}_{ik}(\omega -{\omega }_{ik})\approx {N}_{i}(v).$$

Here, *N*_*i*_(*v*) is the density of ions in the excited state *i* moving with the velocity *v* and the values of frequency and velocity are linked by relation *ω* − *ω*_*ik*_ = *v*/*c*. In the case of an optically thick plasma, it is impossible to measure *N*_*i*_(*v*) directly because the spectral line profile is significantly affected by broadband photoionization absorption.

Ions emitted toward the spectrometer are absorbed less the faster the ions leave the absorbing media. In contrast, ions emitted away from the spectrometer are absorbed more as the distance from the detector increases and the linear density of the absorbing plasma media accordingly rapidly increases with time. Hence, the red-shifted part (long-wavelength wing) of the spectral line profile is strongly depressed by absorption (as can be seen in Figs 1 and 2), while the absorption is insignificant for the far short-wavelength wing of spectral lines. Consequently, even in the case of an optically thick plasma, relation (2) can be applied in particular to the blue-wing part of the line profile to determine the ion distribution.

We note that some depression due to absorption also occurs in the central part of the spectral lines, but it does not affect the spectral line position nor the position of local extremes in the ion distribution function. We consider that the ion distribution function is the same for the ion excited and ground states and use formula (2) to reconstruct the functions *dN*/*dv* and *dN*/*dε* shown in Fig. 2.

### Simulations

To describe ion spectra from a cluster plasma, we use the electrostatic model EGSPC^66^ for the expansion of a single spherical plasma cluster. The input parameters are cold and hot electron temperatures, partial particle densities, and the ion composition. This code allows simulating the adiabatic multi-ion species expansion of a charged or quasineutral spherical plasma cluster for a wide range of the parameter *λ*_*D*_/*D* with homogeneous or inhomogeneous density distributions. This numerical model provides a quick, easy-to-use, and physically crystal clear tool with which we can preliminarily study cluster evolution qualitatively. The aim of the PIC simulations is to improve the fundamental understanding of the cluster expansion mechanism more quantitatively and rigorously. Going beyond the simple electrostatic approach, we also performed PIC simulations of the laser–cluster interaction and cluster expansion in a real 3D geometry using the PIC code Mandor^71^. Clusters were placed in the focal domain of a laser pulse with maximum intensities 10^18^ W/cm^2^ and 3 · 10^17^ W/cm^2^ with the pulse having a Gaussian shape in time (the FWHM duration was 55 fs) and in the transverse direction (the FWHM focal spot size was 4*λ*_L_). The clusters (with initial density profiles shown in Fig. 3a) were composed of electrons, heavy ions with 〈*Z*〉_1_/〈*A*〉_1_ = 1/5, and 1% of light ions with 〈*Z*〉_2_/〈*A*〉_2_ = 1/2. The PIC code used a numerical mesh with a cell size *x* × *y* × *z* = 0.02*λ*_L_ × 0.05*λ*_L_ × 0.05*λ*_L_ and 36 macroparticles of each species per cell.

### Data availability

The data that support the findings of this study are available from the corresponding author upon request.
